# Determining what constitutes an effective psychosocial ‘return to work’ intervention: a systematic review and narrative synthesis

**DOI:** 10.1186/s12889-021-11898-z

**Published:** 2021-11-25

**Authors:** Anthony Venning, Tassia K. Oswald, Jeremy Stevenson, Nicci Tepper, Leva Azadi, Sharon Lawn, Paula Redpath

**Affiliations:** 1 Discipline of Behavioural Health, College of Medicine & Public Health, Flinders University, South Australia, Australia; 2 Remedy HealthCare Group, Melbourne, Victoria Australia; 3 Discipline of Public Health, College of Medicine & Public Health, Flinders University, South Australia, Australia

**Keywords:** Return to work, Intervention, Systematic review, Psychosocial

## Abstract

**Purpose:**

Work can offer a myriad of social and health benefits. Long-term sick leave can be detrimental to employers, individuals, families, and societies. The burden of long-term sick leave has motivated the development of return to work (RTW) interventions. This study sought to determine what constitutes an effective psychosocial RTW intervention, which included exploring whether the level of intervention intensity and intervention characteristics matter to RTW outcomes.

**Methods:**

A systematic review and narrative synthesis were undertaken. Studies were identified through six databases (Ovid Medline, CINAHL (EBSCOhost), PsycInfo (Ovid), ProQuest, Scopus, and Google Scholar) between 2011 and 3 September 2019. Randomised controlled trials (RCTs) or reviews published in English were eligible for inclusion if they targeted adults who were on sick leave/unemployed trying to return to full-capacity employment, had at least one structured psychosocial RTW intervention, and assessed RTW. Study quality was assessed using checklists from the Joanna Briggs Institute.

**Results:**

Database searching yielded 12,311 records. Eighteen RCTs (comprising 42 intervention/comparison groups), seven reviews (comprising 153 studies), and five grey literature documents were included. Included studies were of moderate-to-high quality. Research was primarily conducted in Europe and focused on psychological or musculoskeletal problems. RTW outcomes included RTW status, time until RTW, insurance claims, and sick days. Participating in a RTW program was superior to care-as-usual. RTW outcomes were similar between diverse interventions of low, moderate, and high intensity. Common foundational characteristics seen across effective RTW interventions included a focus on RTW, psychoeducation, and behavioural activation.

**Conclusions:**

Evidence suggests that a low intensity approach to RTW interventions may be an appropriate first option before investment in high intensity, and arguably more expensive interventions, as the latter appear to provide limited additional benefit. More high-quality RCTs, from diverse countries, are needed to provide stronger evidence.

**Supplementary Information:**

The online version contains supplementary material available at 10.1186/s12889-021-11898-z.

## Introduction

Work can offer individuals, families, and wider society a myriad of social and health benefits. From an individual viewpoint, employment can be beneficial for people’s health [1] and contribute to greater quality of life [2], as it affords the opportunity for social integration and contributes to social identity [3]. Most industrialised nations recognise long-term sick leave as an increasing public health problem, with significant personal, social, and economic consequences [4–6]. Currently, common mental health disorders and musculoskeletal problems are the leading cause of absence due to sickness in high-income countries [3, 4, 7, 8] and are associated with both significant personal distress and impairment, alongside public economic burden in the form of lost productivity, wages, and early retirement [3, 9].

Common mental health disorders are the primary driver behind approximately 30 to 50% of all disability claims in high-income countries [9, 10], with the cost of mental ill-health across the Australian workforce estimated to be almost $13 billion in 2015/2016 (approximately $3200 per employee with mental illness) [11]. In Australia, musculoskeletal conditions cause more than 85% of chronic pain and account for over 40% of early retirements, leading to an annual loss of $16 billion in gross domestic product (GDP) [12]. Recognition of the burden associated with long-term sickness absence has motivated policy makers, workplaces, clinicians, and researchers to develop interventions which aim to assist workers to return to work (RTW) and subsequently improving their quality of life. RTW programs respond to the challenge of long-term sickness absence; however, they can be complex and costly [13] and, despite having a shared primary objective of getting people back to work, RTW programs can vary considerably. For example, there is a broad range of interventions in terms of who provides the intervention (e.g., external contractors vs. the workplace themselves) and a broad range of intervention intensities, from low-intensity phone programs to high-intensity supported placements. Furthermore, the contents and methods applied to delivery of RTW interventions can also vary and, at present, no gold standard exists [3, 8].

### The current study

There is growing interest from Australian healthcare providers such as allied health organisations who have the capacity to offer RTW interventions, however it is unclear how these interventions should be designed to best get people back to work. This includes uncertainty around what strategies should be implemented and how intense the level of intervention should be. Through summarising the international literature, the current study aimed to determine what constitutes an effective psychosocial ‘Return to Work’ (RTW) intervention. We define psychosocial interventions as interventions which emphasise psychological, behavioural or social factors rather than biological factors, such as physical health or pharmacotherapy. The current study also aimed to explore whether the level of intervention intensity and intervention characteristics matter to RTW outcomes.

## Methods

### Search strategy

This review drew on the Preferred Reporting Items for Systematic Reviews and Meta Analyses (PRISMA) Guidelines [14]. We systematically searched the following databases between 2011 and 23 July 2019: Ovid Medline, CINAHL (EBSCOhost), PsycInfo (Ovid), ProQuest, Scopus, and Google Scholar. An expert medical librarian helped define the search terms which can be found in Supplementary File 1.

A search of the grey literature was conducted to obtain guidelines and frameworks for RTW best practices that were authored by Australian RTW organisations, given we wanted to address the study aim in the Australian context. The grey literature search involved three strategies recommended by Godin and colleagues [15]. Firstly, an industry expert in the RTW field was contacted for recommendations (a senior manager of a major Australian RTW organisation). This expert provided a list of major RTW organisations in Australia, the websites of which were then searched for relevant documents. An advanced Google search was then conducted using search terms outlined in Supplementary File 1. As recommended, the first 200 Google Scholar results were retained and screened [16].

### Inclusion criteria

Studies were included if they met the following criteria:

- Randomised controlled trials (RCTs) and reviews (systematic or meta-analysis) published in English after 2011 (literature prior to 2011 was reviewed by Hoefsmit, Houkes [1];
- Participants were adults aged 18 years or older on full or partial sick leave, or unemployed, and trying to return to paid employment;
- Interventions focused directly on RTW, or indirectly by addressing a barrier to RTW such as mental illness or pain. At least one of the interventions evaluated in the RCTs, including those in the reviews, needed to be a structured psychosocial intervention that was primarily focused on the individual.
- RTW outcomes were the focus, such as employment status, sickness absence, work-related engagement levels, or disability/insurance claims; or secondary outcomes which generally addressed psychological symptoms such as depression, pain, stress, quality of life, or similar.

High intensity interventions such as work placements, overly medical/physical interventions, intensive case management, interventions with excessive focus on other stakeholders (e.g., meetings with employers or staff training), and interventions with minimal structure (e.g., brokerage case management, in which a case manager provides little direct service to the client), were excluded because we were interested in exploring interventions which could be offered by providers with limited capacity (e.g., small teams, non-clinical settings and resources). Grey literature documents needed to be: (1) authored by an Australian RTW organisation, (2) represent a set of guidelines/frameworks for best RTW practices, (3) allow for inferences about effective intervention characteristics, and (4) be published in English language after 2011.

### Study screening and selection

Two authors (JS and AV) independently screened the titles and abstracts of all articles returned by the systematic search. Where abstracts met the inclusion criteria, the full text was reviewed. Reference lists of identified publications were also checked for additional relevant studies which may have been missed in the database search. The two reviewers met to discuss any discrepancies and agreed upon the final studies for inclusion.

### Critical appraisal of included studies

The included studies were critically appraised using checklists from the Joanna Briggs Institute for RCTs or reviews. The checklists assess the quality of each study’s methodology, referring to important elements of study. Two authors (JS and TKO) independently appraised the studies. When study ratings were incongruent, the two authors discussed until consensus was reached. A higher percentage score indicates higher quality methodology. Studies with a score of 50% or less were excluded from the review.

### Data extraction and synthesis

Data were extracted and cross-checked from the included studies by a combination of three review authors (JS, TKO and AV) using data collection tools designed and tested by the review authors.

General study characteristics and results extracted from included RCTs included study location, population, sample size, intervention details, intervention provider(s), RTW outcome(s), and secondary outcome(s). Information about common intervention characteristics were identified and are described in Table 1.

**Table 1:** RCT Intervention Characteristics: Description and Categorisation

Intervention Characteristic	Categorisation	Description
Description of Intervention	N/A	Main components of intervention
Duration (Total Hours)	Low / Moderate / High	Low ≤4 / Moderate > 4 < 12 / High ≥12
Frequency	Low / Moderate / High	Low ≤ monthly / Moderate > monthly < weekly / High ≥ weekly
Early Timing*	Yes/No	Whether the intervention occurred within 3 months of initial sickness absence
Multi-disciplinary	Yes/No	Whether intervention was multi-disciplinary
RTW focus	Yes/No	Whether the intervention had some explicit focus on return to work
Exposure	Yes/No	Whether the intervention had some focus on external (e.g., graded RTW) or internal (e.g., mindfulness) exposure (i.e., confrontation of challenging stimuli)
Cognitive Restructuring	Yes/No	Whether the intervention included a focus on cognitive restructuring
Behavioural Activation	Yes/No	Whether the intervention had a focus on behavioural activation (e.g., exercise, remaining active, activity scheduling)
Goal Setting	Yes/No	Whether the intervention included a focus on goal setting
Values Clarification	Yes/No	Whether the intervention included a focus on value clarification or identification
Problem Solving	Yes/No	Whether intervention included a focus on problem solving (e.g., identification of barriers and strategies to overcome these)
Psychoeducation	Yes/No	Whether intervention included a focus on psychoeducation

* = consistent with a previous definition by Loisel and colleagues [17]

At times, a secondary reference was located (e.g., the RCT protocol) to gain more detailed information about intervention characteristics. On some occasions, assumptions were made; for example, if a study included a common intervention (e.g., Cognitive Behavioural Therapy), but did not specify the length of each session, it was deemed reasonable to assume that sessions were one hour each, as this is the average duration of an individual psychotherapy session [18].

For the included reviews, data extracted included type of review, objective, study types, participants, and the main findings. For grey literature, data collected included: author, year of publication, title, target population, and relevant recommendations.

Three steps of narrative synthesis, as outlined by Popay and colleagues [19], were followed: (1) develop a preliminary synthesis, (2) explore relationships in the data, and (3) assess the robustness of the synthesised product. In order to develop a preliminary synthesis of findings, the data were (a) organised into groups relating to study design, type of intervention, and setting or context for the intervention; and (b) presented in tabular form.

## Results

### Study search and inclusion

**Fig. 1:**
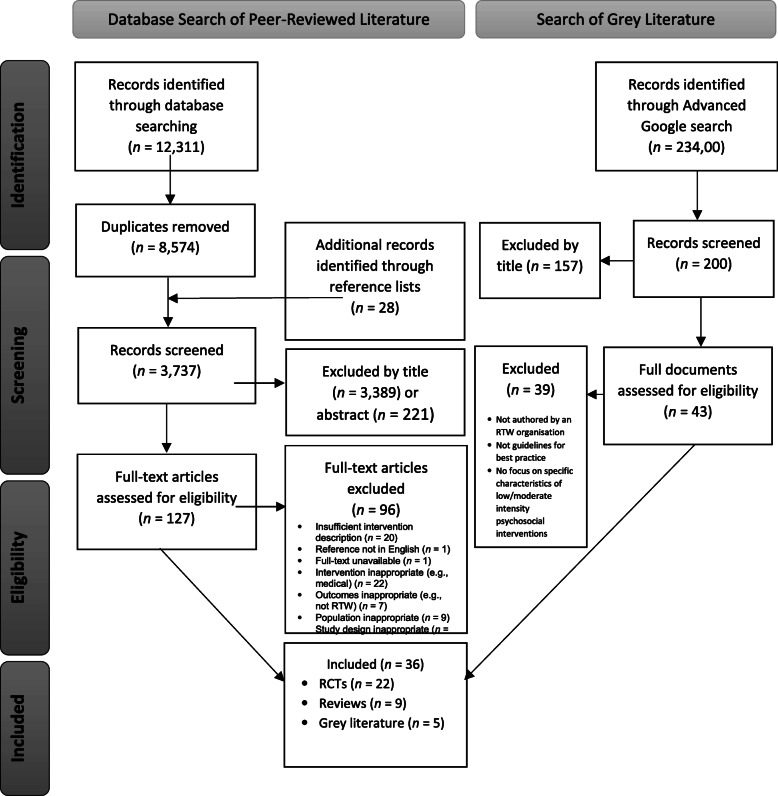
PRISMA Flow-chart of study search and selection. *N/A = not applicable*

As shown in Fig. 1, database searching yielded 12,311 records. Following the removal of duplicates, 3737 studies were screened by title and abstract, and 3610 studies were excluded. Reference list searching resulted in the identification of an additional 28 relevant studies and the full text of 127 studies were assessed. Thirty-one studies were included, comprising of 22 RCTs and 9 reviews.

For the grey literature, the industry expert suggested several major RTW organisations and websites. However, a search of these organisations websites did not yield any results. An advanced Google search occurred on the 3rd of September 2019. As shown in Fig. 1, the advanced Google search returned 234,000 results. The first 200 results were screened and 157 were excluded by title. Forty-three full documents were reviewed, and 5 documents were subsequently included.

### Quality of included studies

**Fig. 2:**
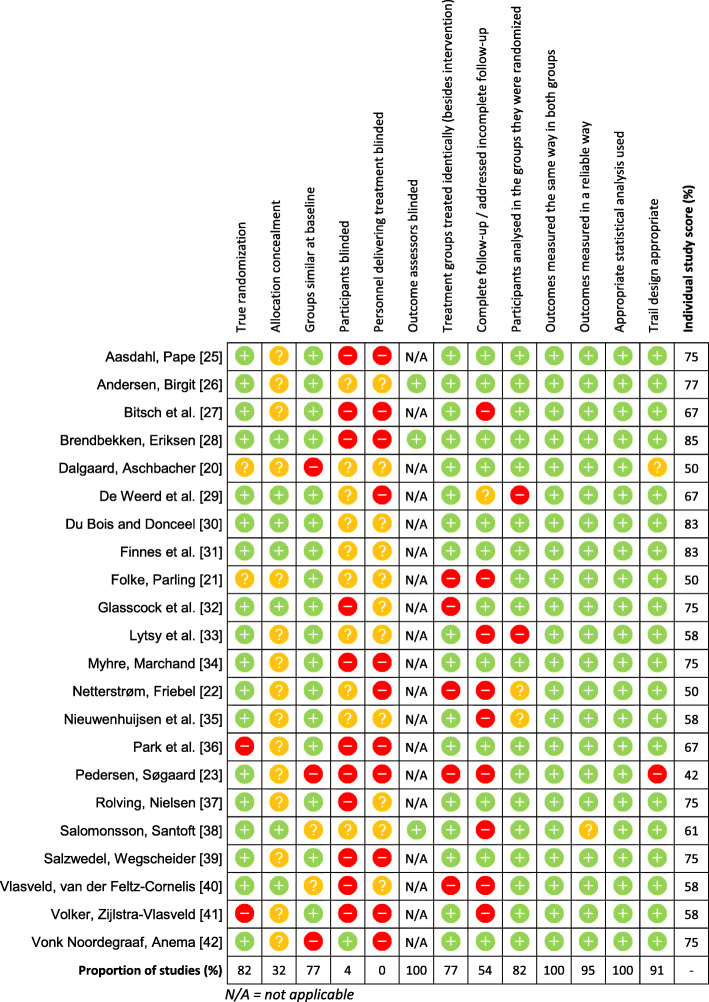
Critical appraisal of included randomized controlled trials*. N/A = not applicable*

Critical appraisal of the included RCTs (*n* = 22) is displayed in Fig. 2. Four included RCTs [20–23] were removed from the systematic review because they were classified as low quality (appraisal scores ≤50%). Appraisal scores for the remaining 18 RCTs ranged between 58 and 85%. Randomisation procedures, statistical analyses performed, trial designs utilised, and measurement of outcomes for intervention and control participants were done appropriately in the majority of the included RCTs.

**Fig. 3:**
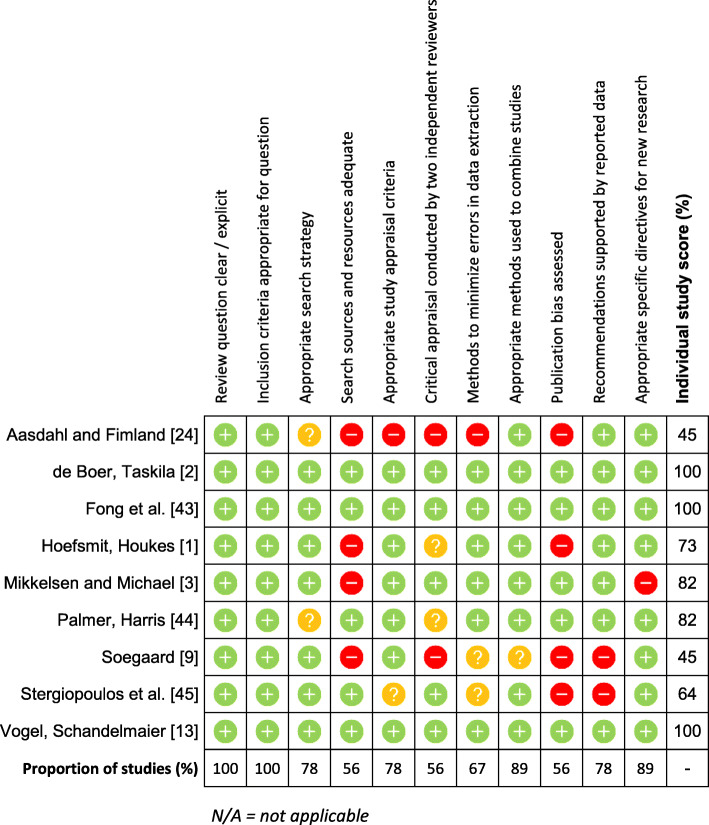
Critical appraisal of included reviews

Most studies utilised administrative or registry data, removing the possibility of assessment bias. The biggest limitation across the majority of the included RCTs was an inability to determine whether the participants themselves, or those delivering the interventions, were blinded. Furthermore, descriptions and considerations of participants lost to follow-up were typically inadequate. Critical appraisal of the included reviews (*n* = 9) is displayed in Fig. 3. Two included reviews [9, 24] were removed from the systematic review because they were classified as low quality (45%). Appraisal scores for the remaining 7 reviews ranged between 64 and 100%, with three receiving a score of 100%. The main methodological limitations in the included reviews included failing to include grey literature in searches, not using two reviewers to independently appraise studies, and not formally assessing publication bias. The remainder of the checklist items were met by the majority of the reviews.

### Randomised controlled trials (*n* = 18)

#### General study characteristics

General characteristics of the included RCTs are shown in Table 2. Eighteen RCTs were included in the review, comprising of 42 intervention/comparison groups. All RCTs were conducted in central or northern Europe, with one study conducted in Canada. Total sample sizes ranged from 60 to 728 participants (Median (IQR) = 217.50 (218.25)). Target populations included individuals with musculoskeletal problems (*n* = 8 studies; 44.5%), psychological problems (*n* = 9 studies; 50%), and other health issues (*n* = 3 studies; 17%). Only 3 studies (17%) explicitly stated that the health problems were work-related. Participants in the included RCTs had mixed employment status (casual, part-, or full-time). In most studies, participants’ sick leave duration was long (> 3 months) and generally up to 12 months.

**Table 2:** General characteristics of included RCTs (*n* = 18)

Study	Country	***n***	Target Population	Intervention 1		Intervention 2		RTW Measure(s)	Secondary Outcome(s)
				Description	Provider(s)	Description	Provider		
**Aasdahl, Pape** **[**25**]**	Norway	168	Adults with musculoskeletal or mental health disorders, on 50–100% sick leave for 2–12 months	Outpatient group-based ACT in addition to 2 individual sessions to clarify personal values and work-related issues; homework assigned between sessions including daily 15-min mindfulness practice; group-based motivational discussion about benefits of physical training; concluding with an individual session to write a letter to GP	Physician, psychologist, social worker, physiotherapist	Inpatient multi-component intervention including group ACT, physical training, mindfulness, psychoeducation, problem solving, RTW plan creation	Mentors / coordinators, rehabilitation center staff	Number of sickness absence days and time until RTW (1 month without relapse)	None
**Andersen, Birgit** **[**26**]**	Denmark	141	Adults with pain in back or upper body, mixed employment status, on sick leave for a maximum of 9-weeks	Health guidance dialogue about lifestyle, motivation, resources, power to act; opportunity to create health plan for improving daily wellbeing	Health supervisor	Health guidance + tailored physical activity: group fitness and strength training *OR*	Health supervisor, physiotherapists	RTW status	Pain; BMI; aerobic capacity; work ability; kinesiophobia
						Health guidance + chronic pain self-management including group-based problem solving, exercise, and psychoeducation	Health supervisor, trained non-health professional facilitators		
**Bitsch, Nielsen** **[**27**]**	Denmark	244	Adults with ischemic heart disease or heart failure undergoing cardiac rehabilitation	Standard cardiac rehabilitation involving structured deductive teaching (pre-written slideshows) without rationale; education topics included lifestyle implications, emotional reactions, importance of social networks, importance of exercise, future life with chronic disease	Nurse, physiotherapist, experienced patients	Learning and coping (LC) education program in addition to standard cardiac rehabilitation; inductive learning with rationale; 2 individual clarifying interviews	Nurse, physiotherapist, experienced patients, LC trained health professionals	RTW status and relapse (RTW, but not at work 1 year later)	None
**Brendbekken, Eriksen** **[**28**]**	Norway	284	Adults with musculoskeletal pain, on 50–100% sick leave for less than 12 months	Brief intervention focusing on education, reduction of fear and concern, helping patient stay active; opportunity for patient to express problems and worries; thorough medical and educational examination	Physician, physiotherapist	Multi-disciplinary comprehensive intervention involving multiple interviews and assessments, visual feedback tools	Social worker, physician, physiotherapist	Full-time RTW status	None
**De Weerd, Van Dijk** **[**29**]**	Netherlands	60	Adults with common psychological disorders, employed and on part-time sick leave	Work-focused CBT focusing on activation, day structure, socialising, and self-worth within a work context	Provisional and registered psychologists	Work-focused CBT plus a 1.5 h convergence dialogue meeting between patient, employer, and psychologist discussing RTW	Provisional and registered psychologists, supervisor	Time until first and full RTW	Mental health
**Du Bois and Donceel** **[**30**]**	Belgium	506	Adults with low back pain, employed and on sick leave	Rehabilitation-oriented coaching involving medical and psychosocial education, importance of activity, response rates, and encouragement based on resilience	Physician	CAU involving brief disability evaluation without medical advice	Unclear	RTW status; sick leave recurrence; sick leave duration	Subsequent surgery
**Finnes, Ghaderi** **[**31**]**	Sweden	352	Adults with common psychological disorders, employed and on 25–100% sick leave for previous 1–12 months	ACT alone involving some work focus, psychoeducation about avoidance patterns, mindfulness, diffusion, self-compassion, value-driven behaviour, exposure exercises	Psychologists	WDI: three-steps, to facilitate dialogue between participant and workplace *OR* WDI + ACT *OR* TAU: saw medical doctors, psychologists, social workers, physical therapists, and nurses in the study period.	Supervisor, psychologists, medical doctors, social workers, physical therapists, and nurses	Number of sickness absence days	Work ability; general functioning; exhaustion; depression; anxiety
**Glasscock, Carstensen** **[**32**]**	Denmark	137	Adults with work-related adjustment, stress or mild depression, employed and on sick leave for < 4 months	Individual work-focused CBT; psycho-education; establishing shared treatment goals; stress coping; cognitive restructuring; relapse prevention; workplace intervention involving meeting with employers with option of psychologist attendance	Psychologists	No-treatment control group	N/A	Lasting RTW (> 4 weeks)	Stress; general health
**Lytsy, Carlsson** **[**33**]**	Sweden	308	Adult females with pain or mental illness, mixed employment status and about to reach maximum sick leave duration of 1 year	ACT focused on behavioural strategies to increase function and quality of life, rather than symptom reduction	Psychologists	Multidisciplinary assessments, creation of individualised rehabilitation plans, and weekly meetings to evaluate / synchronise plans and activities. ACT was an option where suggested by the team. *OR* “Usual care” by regular health contacts	Physician, psychologist, occupational therapist, social worker.	Insurance status; insurance days; working hours; work engagement	None
**Myhre, Marchand** **[**34**]**	Norway	405	Adults with neck and back pain, employed and on sick leave for 1–12 months	Pain-related coaching: standard clinical examination; education about importance of activity; focus on reduction in fear-avoidance beliefs; self-care and coping	Physician, physiotherapist	Pain-related coaching plus appointments with caseworker focusing on RTW, opportunity for a meeting with employer	Physician, caseworker, physiotherapist	Lasting absence (5 weeks) of welfare benefits	None
**Nieuwenhuijsen, Antonius** **[**35**]**	Netherlands	96	Adults with work-related chronic stress (neurasthenia), employed and on part- or full-time sick leave	RTW and mental health coaching focused on reducing burnout and improving wellbeing; problem-solving related to mental health; psycho-education; cognitive restructuring; acceptance and relaxation; employability; conflict and time management; fatigue and stress	Certified coach	RTW and mental health coaching plus light / electromagnetic field therapy *OR* RTW and mental health coaching plus placebo light / electromagnetic therapy	Certified coach	Percentage RTW	Emotional exhaustion; fatigue; quality of life; stress; cortisol
**Park, Esmail** **[**36**]**	Canada	728	Adults with work-related musculoskeletal injuries, mixed employment status	Motivational interviewing focused on enhancing motivation, reducing ambivalence, eliciting reasons for change, discussing ability to change	Case managers, physicians, psychologist, occupational therapists, exercise therapists	CAU involving functional restoration – an interdisciplinary approach that focuses on improving physical and functional abilities, RTW planning, and individual counselling and educational workshops	Various stakeholders, occupational therapists, exercise therapists	RTW status	None
**Rolving, Nielsen** **[**37**]**	Denmark	90	Adults with degenerative disc disease or spondylolisthesis receiving lumbar spinal fusion surgery, mixed employment status	Group-based and standardised preoperative CBT focusing on the relationship between thoughts and pain, cognitive restructuring, coping, pacing, RTW, ergonomics, surgery information, pleasant activity scheduling, problem-solving	Psychologist, occupational therapist, physiotherapist, social worker, spine surgeon, previous patient	CAU involving standard preoperative surgery-related information and postoperative physical rehabilitation	Operating surgeons, nurses, physiotherapists, occupational therapists	RTW status	Disability; fear avoidance; catastrophizing; pain
**Salomonsson, Santoft** **[**38**]**	Sweden	211	Adults with common psychological disorders, on 50–100% sick leave for 1–6 months	Psychological disorder-focused treatment. CBT involving a protocol for the primary diagnosis (e.g., behavioural activation for depression, cognitive therapy for social phobia, exposure for obsessive-compulsive disorder)	Psychologists	RTW-focused CBT only *OR* Psychological disorder and RTW-focused CBT combined	Psychologists	Number of sick leave days	Psychological symptoms; work ability; quality of life; stress
**Salzwedel, Wegscheider** **[**39**]**	Germany	354	Adults with recent acute cardiac event and negative RTW expectations, mixed employment status, unemployed for less than 1 year	Group-based social counselling and therapy focused on legal rights, occupational capacity and health behaviour, promoting social competency such as networking	Social workers	CAU involving cardiac rehabilitation and as-needed counselling	Social workers	RTW status	Quality of life
**Vlasveld, van der Feltz-Cornelis** **[**40**]**	Netherlands	126	Adults with major depressive disorder, on sick leave for 1–3 months	Collaborative care involving structured problem-solving; self-help focused on cognitive restructuring, RTW, and healthy lifestyle; workplace intervention; possible prescription of anti-depressants	Occupational physician, psychiatrist	CAU involving contact with a range of health professionals and treatment for mental health problems in some cases	Occupational physician, GP, mental health professionals, social worker, medical specialist, paramedic, alternative healthcare, self-help group	Time until full RTW	Depression
**Volker, Zijlstra-Vlasveld** **[**41**]**	Netherlands	220	Adults with common psychological disorder, on sick leave for 1–6 months	Web-based blended individually tailored eHealth including several of the following modules: 1) psycho-education, 2) CBT for RTW-related symptoms, 3) problem-solving, 4) pain and fatigue management and reactivation, 5) relapse prevention; consultations with occupational physician	Occupational physician	CAU involving access to various health professionals; regular consultations with occupational physician	Occupational physicians, general practitioner, mental health professional, social worker, self-help group	Time until first RTW; time until full RTW; number of sick leave days	Psychological symptoms
**Vonk Noordegraaf, Anema** **[**42**]**	Netherlands	215	Adult females with hysterectomy and/or laparoscopic adnexal surgery, employed and on sick leave for less than 2 months	Web-based tailored eHealth including pre/post-operative instructions on RTW and daily activities; self-empowerment; communication with stakeholders; identification of recovery barriers; general information on the surgery; frequently asked questions; forum to communicate with other participants	None	Web-based control with generic information related to the surgery	Usual care given by gynaecologists, occupational physicians, and general practitioners	Time until full RTW	Quality of life; general recovery; pain

*Note*: *n* number of participants, *RTW* return to work, *ACT* Acceptance and Commitment Therapy, *CBT* Cognitive Behavioural Therapy, *GP* general practitioner, *CAU* care as usual, *WDI* workplace dialogue intervention, *GP* general practitioner; ^a^With pre-planned adjusted analyses

Interventions in the included RCTs were typically well-known structured psychosocial interventions, including Cognitive Behaviour Therapy (CBT) and Acceptance and Commitment Therapy (ACT). Other interventions such as coaching and counselling were also used. Four RCTs involved a workplace mediation component and only two interventions involved web-based engagement. Common RTW outcome measures included RTW status (i.e., succeeded or failed in returning to work), time until first or full RTW, disability/insurance claims, and number of sick leave days. Most studies also included secondary outcome measures which generally addressed psychological symptoms such as depression, stress, quality of life, and pain.

#### Overall study results

Table 3 presents specific intervention details (including intervention duration, frequency, and psychological strategies used), as well as results from the included RCTs. There were largely no statistically significant differences on RTW outcomes between participants in diverse RTW interventions across the RCTs. Participants generally had positive outcomes and there was no indication that any of the interventions were detrimental to an individuals’ RTW (see Table 3). Eleven of the included RCTs reported on participants RTW outcomes at approximately 1-year follow-up [25–28, 30, 34, 36, 37, 39–41]. The percentage of participants who achieved RTW at 1-year follow-up in these studies varied considerably between and within studies. On average, the average minimum proportion of participants who achieved RTW at 1-year follow-up within these studies was 56%, while the average maximum proportion of participants who achieved RTW at 1-year follow-up within these studies was 68% of participants.

**Table 3:** Specific Intervention Characteristics & Overall Results from Included RCTs (*n* = 18)

Study	Interventions	Duration (Total Hours)	Frequency of sessions	Early Timing	Multi-Disciplinary	RTW Focus	Exposure	Cognitive Restructuring	Behavioural Activation	Goal Setting	Values Clarification	Problem Solving	Psychoeducation	Results
**Aasdahl, Pape** **[**25**]**	Outpatient group-based ACT	High (18)	High (Weekly)	**✕**	**✓**	**✓**	**✓**	**✕**	**✓**	**✓**	**✓**	**✕**	**✓**	• NSSD between groups in sickness absence days 6 or 12 months following the programs. • NSSD between groups in sustainable RTW (1 month without benefits) at 12 months follow-up (OP = 57%; IP = 49%).
	Inpatient multi-component intervention	High (56)	High (4 days over 2 weeks)	**✕**	**✓**	**✓**	**✓**	**✕**	**✓**	**✓**	**✓**	**✓**	**✓**	
**Andersen, Birgit** **[**26**]**	Health guidance	Low (1.5)	Low (One-off)	**✓**	**✕**	**✕**	**✕**	**✕**	**✓**	**✕**	**✕**	**✕**	**✕**	• NSSD’s between intervention types in RTW after 11 months (HG = 64%; TPA = 61%; CPSM = 60%). • Pain, BMI, aerobic capacity, work ability and kinesiophobia improved, and non-significant changes were found between groups.
	Tailored physical activity + Health guidance ***OR*** Chronic pain self-management + Health guidance	High (> 30)	Highly (weekly)	**✓**	**✓**	**✕**	**✕**	**✕**	**✓**	**✕**	**✕**	**✕**	**✕**	
		High (16.5)	High (weekly)	**✓**	**✓**	**✕**	**✕**	**✕**	**✓**	**✕**	**✕**	**✓**	**✕**	
**Bitsch, Nielsen** **[**27**]**	Standard cardiac rehabilitation alone involving structured deductive teaching without rationale and 3 exercise sessions per week	High (48)	High (4 per week)	**✓**	**✓**	**✕**	**✕**	**✕**	**✓**	**✕**	**✕**	**✕**	**✓**	• NSSD between groups in successful RTW at 1-year follow-up (SCR = 69%; LC = 65%). • NSSD in relapse rates between the groups.
	Learning and coping education program in addition to standard cardiac rehabilitation	High (50)	High (4 weekly + 2 interviews)	**✓**	**✓**	**✕**	**✕**	**✕**	**✓**	**✕**	**✕**	**✕**	**✓**	
**Brendbekken, Eriksen** **[**28**]**	Brief intervention focusing on education	Low (3.5)	Moderate (Fortnightly)	**✕**	**✓**	**✕**	**✕**	**✕**	**✓**	**✕**	**✕**	**✕**	**✓**	• NSSD between groups in full RTW at 12- (45% for both) and 24- (BI = 37%; MI = 43%) months follow-up. • In three of the first 7 months, significantly more patients were partly RTW in the MI-group compared to the BI-group (RR = 2.31 (95% CI 1.19–4.51, *p* = 0.01)).
	Multi-disciplinary comprehensive intervention	Moderate (5.5)	Moderate (Fortnightly + 3-month follow-up)	**✕**	**✓**	**✓**	**✕**	**✕**	**✓**	**✕**	**✕**	**✓**	**✓**	
**De Weerd, Van Dijk** **[**29**]**	Work-focused CBT	Moderate (6)	High (Weekly)	**✓**	**✕**	**✓**	**✓**	**✓**	**✓**	**✕**	**✕**	**✕**	**✓**	• NSSD between groups in first RTW at the end of treatment (CBT = 26 participants; CA = 29 participants). • NSSD between groups in full RTW at the end of treatment (17 participants in each). • NSSD between groups on the Symptom Checklist-90 items after treatment. • Some effect modification by gender.
	Work-focused CBT plus a 1.5 h meeting with employer	Moderate (7.5)	High (Weekly)	**✓**	**✕**	**✓**	**✓**	**✓**	**✓**	**✕**	**✕**	**✕**	**✓**	
**Du Bois and Donceel** **[**30**]**	Rehabilitation-oriented coaching	?	Low (Monthly)	**✕**	**✕**	**✓**	**✕**	**✕**	**✓**	**✕**	**✕**	**✕**	**✓**	• At 1-year follow-up, 8% of participants in the brief disability evaluation group had not returned to work, compared with 4% in the coaching group (p = 0.03). • At 1-year follow-up recurrent sick leave was higher in the no medical advice group (23.3%) compared to the coaching group (15.3%) (*p* = 0.02). • NSSD between the groups regarding subsequent surgery for lower back pain or duration of sick leave.
	Brief disability evaluation without medical advice	Low (1)	Low (once-off)	**✕**	**✕**	**✕**	**✕**	**✕**	**✕**	**✕**	**✕**	**✕**	**✕**	
**Finnes, Ghaderi** **[**31**]**	ACT	Moderate (6)	Moderate (Fortnightly)	**✕**	**✕**	**✓**	**✓**	**✕**	**✓**	**✓**	**✓**	**✕**	**✓**	• Overall, net sickness absence days decreased by approximately 16 days from pre- to post-treatment, but there was NSSD between groups (some group differences during follow-up when stratified by diagnostic group). • All groups improved in self-assessed work ability (WAI) from pre- to post-measurement, but there was NSSD between groups (some group differences during follow-up when stratified by diagnostic group). • For general functioning (WSAS), there was NSSD between groups for pre- to post-measurement or during follow-up. • For satisfaction with life (SWLS), there was a significant Group x Time effect from pre- to post-measurement. The participants randomized to ACT and WDI improved significantly more than TAU. The ACT + WDI condition did not differ from TAU. For the follow-up period, there were no differences between groups. • There were some differences in symptom reduction between groups at post-treatment, favouring mostly ACT and ACT + WDI, but there were no differences in overall estimated average linear change between groups during the follow-up period for any of the secondary outcome measures. These results indicate that up until 9-month follow-up, self-reported symptoms of depression, anxiety, and exhaustion disorder decreased, whereas daily functioning and satisfaction with life increased over time, but changes were similar across conditions.
	WDI ***OR***	Low (2)	Low (2 interviews / meetings)	**✕**	**✕**	**✓**	**✕**	**✕**	**✕**	**✕**	**✕**	**✓**	**✕**	
	WDI + ACT ***OR***	Moderate (9)	Moderate (fortnightly)	**✕**	**✓**	**✓**	**✓**	**✕**	**✓**	**✓**	**✓**	**✓**	**✓**	
	TAU involving rehabilitation in standard care facilities with a range of health professionals	**?**	**?**	**✕**	**?**	**?**	**?**	**?**	**?**	**?**	**?**	**?**	**?**	
**Glasscock, Carstensen** **[**32**]**	Work-focused CBT with option of workplace meeting	Moderate (6)	High (Weekly)	**✕**	**✕**	**✓**	**✓**	**✓**	**✓**	**✓**	**✕**	**✕**	**✓**	• NSSD between groups for sick-leave duration or lasting RTW. • At 10-month follow up, both groups reported less perceived stress and improved mental health, but there were NSSDs.
	No-treatment control group	**✕**	**✕**	**✕**	**✕**	**✕**	**✕**	**✕**	**✕**	**✕**	**✕**	**✕**	**✕**	
**Lytsy, Carlsson** **[**33**]**	ACT	High (no limit)	High (Weekly)	**✕**	**✕**	**✕**	**✓**	**✕**	**✓**	**✓**	**✓**	**✕**	**✓**	• NSSD’s between groups for returning to health insurance system (CAU = 51.5%; ACT = 43.5%; MD = 39%) or number of reimbursed days from the healthcare system (12-month follow up). • Compared to the “usual care” group (30%), participants in the multidisciplinary intervention (51%, *p* = 0.015) were more likely to self-report increased work-related degree of engagement, but ACT (40%, *p* = 0.21) participants were not. • Compared to the “usual care” group, participants in the multidisciplinary intervention were more likely to report increased working hours (OR 2.20 (95% CI 1.09–4.44, *p* = 0.028)), but ACT participants were not (OR of 0.95 (95% CI 0.46–1.95, *p* = 0.90)).
	Multidisciplinary assessments and individual rehabilitation interventions ***OR***	High (no limit)	Moderate (approx. weekly)	**✕**	**✓**	**✓**	**?**	**?**	**?**	**?**	**?**	**?**	**?**	
	“Usual care” provided by regular health contacts	?	?	**?**	**?**	**?**	**?**	**?**	**?**	**?**	**?**	**?**	**?**	
**Myhre, Marchand** **[**34**]**	Pain-related coaching	High (20)	High (Weekly)	**✕**	**✓**	**✓**	**✕**	**✓**	**✓**	**✕**	**✕**	**✕**	**✓**	• NSSD’s between groups for RTW within 12 months (pain-related coaching only = 75%; pain-related coaching + work-focus = 70%) and median time before RTW (pain-related coaching only = 158 days; pain-related coaching + work-focus = 161 days). • In subgroup analyses, the median time before RTW was significantly briefer in the pain-related coaching only group than in the work-focus group, for participants aged > 41 years (132 vs. 177 days, *p* = 0.03).
	Pain-related coaching plus caseworker focusing on RTW and opportunity for a meeting with employer	High (appx. 20)	High (Weekly)	**✕**	**✓**	**✓**	**✕**	**✓**	**✓**	**✕**	**✕**	**✕**	**✓**	
**Nieuwenhuijsen, Antonius** **[**35**]**	RTW and mental health coaching only	Moderate (6)	Moderate (Fortnightly)	**?**	**✕**	**✓**	**✕**	**✓**	**✕**	**✕**	**✓**	**✓**	**✓**	• NSSD’s between groups on all outcome measures. • Over time, participants in all groups increased their working hours to approximately two-thirds of their contract hours on average. • Emotional exhaustion complaints and stress complaints significantly decreased after treatment in all groups. • Work-related fatigue levels decreased significantly after treatment in all groups, to about half the level it was when the study started. • Significant improvements in QOL were observed over time for all groups.
	Light therapy / electromagnetic field therapy plus RTW and mental health coaching **OR**	Moderate (6)	Moderate (Fortnightly)	**?**	**✕**	**✓**	**✕**	**✓**	**✕**	**✕**	**✓**	**✓**	**✓**	The results from the box above can be incorporated with this blank box, as they apply to the one study - Nieuwenhuijsen, Antonius
	RTW and mental health coaching plus placebo light / electromagnetic therapy	Moderate (6)	Moderate (Fortnightly)	**?**	**✕**	**✓**	**✕**	**✓**	**✕**	**✕**	**✓**	**✓**	**✓**	
**Park, Esmail** **[**36**]**	Functional restoration plus motivational interviewing	?	?	**✕**	**✓**	**✓**	**✓**	**✕**	**✓**	**✓**	**✓**	**✕**	**✕**	• Successful RTW at program discharge was 12.1% higher for unemployed claimants in the MI group overall (21.6%) vs. the CAU group (9.5%) (*p* = 0.03). • Successful RTW was also 3% higher for job attached claimants in the MI group (97.1%) compared to the CAU group (94.1%) (*p* = 0.10). • The proportion of claimants with successful RTW in the MI adherent intervention group was 33.3%, higher than the non-adherent intervention group (18.0%) and control group (9.5%) (*p* < 0.01). • Successful RTW increased to 47.4% when the MI adherent intervention included RTW as the target behaviour. • RTW among those who were job attached was higher in the MI adherent group (100%) compared to the non-adherent MI group (96.3%) and the control group (94.1%) (p – 0.03).
	CAU involving functional restoration	?	?	**✕**	**✓**	**✓**	**✓**	**?**	**✓**	**?**	**?**	**?**	**?**	
**Rolving, Nielsen** **[**37**]**	Preoperative CBT in addition to CAU	High (18)	High (Weekly)	**✓**	**✓**	**✓**	**✓**	**✓**	**✓**	**✕**	**✕**	**✓**	**✓**	• NSSD between the groups RTW rate during the first year after surgery (CBT = 42%; CAU = 42%) or sick leave (mean weeks) during the first year after surgery (CBT = 31; CAU = 39). • NSSD between the groups’ disability (ODI) scores at 12-month follow-up. At 3-months follow-up the CBT group achieved better. • NSSD between the groups for measures of catastrophizing or fear avoidance belief at 12-month follow up. However, at 6-months follow up the CBT group achieved better. • NSSD between the groups in terms of reduction in back / leg pain during the first year after surgery.
	CAU involving standard preoperative surgery-related information and postoperative physical rehabilitation	Moderate (appx 8)	High (Weekly)	**✕**	**✓**	**✕**	**✕**	**✕**	**✓**	**✕**	**✕**	**✕**	**✕**	
**Salomonsson, Santoft** **[**38**]**	Psychological disorder focused CBT (non-work focused)	High (maximum of 20 sessions)	High (Weekly)	**✕**	**✕**	**✕**	**✓**	**✓**	**✓**	**✕**	**✕**	**✕**	**✓**	• NSSD between groups for days of sick-leave at 12-months follow-up. • NSSD between groups for proportion of patients on full-time sick leave, part-time sick leave, or without sick-leave at 6- and 12-month follow up (no longer on sick leave at 12 months follow-up: CBT = 77%, RTW-I = 79%; COMBO = 80%). • Pre- to post-treatment, there were significant differences between groups on the clinician’s severity rating (CSR) outcome. Results indicated superior reduction of psychiatric symptoms after CBT compared with RTW-I. There was NSSD between COMBO and the other treatments pre to post. From post-treatment to 12-months follow up, RTW-I led to a larger improvement on the CSR compared with CBT. COMBO did not differ from CBT or RTW-I post-treatment to 12-months follow up. Within-group effect sizes (d’s) were large pre- to post-treatment for all groups; CBT 2.5 (95% CI 1.8 to 3.2), COMBO 2.0 (95% CI 1.5 to 2.4) and RTW-I 1.6 (95% CI 1.2 to 2.1). • 67% of the total sample did not fulfil criteria for principal disorder at post-treatment. At 12-months follow up, 26% of patients reported an increase in psychotropic medication or additional psychological treatment (NSSDs between groups).
	RTW-focused CBT only ***OR***	Moderate (10)	Moderate (fortnightly)	**✕**	**✕**	**✓**	**✓**	**✕**	**✓**	**✓**	**✕**	**✓**	**✓**	
	Psychological disorder and RTW-focused CBT combined	High (maximum of 25 sessions)	High(Weekly)	**✕**	**✕**	**✓**	**✓**	**✓**	**✓**	**✓**	**✕**	**✓**	**✓**	
**Salzwedel, Wegscheider** **[**39**]**	Social counselling and therapy in addition to usual care (cardiac rehabilitation)	Moderate (6)	High (Twice weekly)	**✓**	**✕**	**✓**	**✓**	**✕**	**✓**	**✓**	**✕**	**✓**	**✕**	• NSSD between the groups for employment 12 months after cardiac rehabilitation (social counselling / therapy group = 42.1%; CAU = 45.1%). • NSSD’s between the groups in sick leave, disability pension, or change in occupational situation. • NSSD between groups on measures of work ability (WAI) or mental quality of life.
	CAU involving cardiac rehabilitation and as-needed counselling	?	?	**✓**	**✕**	**?**	**?**	**?**	**?**	**?**	**?**	**?**	**?**	
**Vlasveld, van der Feltz-Cornelis** **[**40**]**	Collaborative care involving problem-solving, manual-guided self-help, workplace intervention, possible anti-depressants	High (max 12 sessions)	?	**✓**	**✓**	**✓**	**✕**	**✓**	**✕**	**✕**	**✕**	**✓**	**✓**	• At 12-months follow up, 65% of the collaborative care and 59% of the CAU participants achieved lasting, full RTW. • NSSD in groups on duration until lasting, full RTW (collaborative care group = 190 (SD 120) days; CAU = 210 (SD 124) days (*p* > 0.05). • NSSD between groups on mean number of sick days in the entire follow up period (collaborative care group = 198 (SD 120) days; CAU = 215 (SD 118) days) (*p* > 0.05). • NSSD between groups on the PHQ-9 at all follow-up points. • Compared with the collaborative care group, more participants in CAU group had received treatment for mental health problems (14.5% vs 1.8%).
	CAU involving contact with a range of health professionals and treatment for mental health problems in some cases	?	?	**✓**	**?**	**?**	**?**	**?**	**?**	**?**	**?**	**?**	**?**	
**Volker, Zijlstra-Vlasveld** **[**41**]**	Blended eHealth individually tailored involving CBT and problem-solving; access to face-to-face meetings with occupational physician	?	?	**✕**	**✕**	**✓**	**✕**	**✓**	**✓**	**✕**	**✕**	**✓**	**✓**	• By 1-year follow-up, 84% (72/86) of the CAU participants and 87.7% (114/130) of the e-Health participants had achieved partial or full RTW. • Duration until first RTW differed between groups. The median duration was 77 (CAU group) and 50 days (eHealth group) (*p* = .03). NSSD was found between the groups for duration until full RTW. • NSSD in the median total number of sick days in the 1-year follow-up period (CAU = 228.0 days (IQR 111.0–365.0); e-Health = 174.0 days (IQR 100.0–321.0)). • NSSD between groups for common mental disorder symptoms, but at 9 months follow up significantly more participants in the eHealth group (*n* = 41; 56%) achieved remission than in the CAU group (*n* = 23; 37%) (*p* = .02).
	CAU involving access to various health professionals; regular consultations with occupational physician	?	?	**✕**	**?**	**?**	**?**	**?**	**?**	**?**	**?**	**?**	**?**	
**Vonk Noordegraaf, Anema** **[**42**]**	Web-based tailored eHealth focused on recovery and RTW	Low (maximum 3 h)	?	**✓**	**✕**	**✓**	**✕**	**✕**	**✓**	**✕**	**✕**	**✕**	**✕**	• There was a statistically significant difference in RTW, favoring the treatment group focusing on recovery and RTW (HR = 1.54, 95%CI 1.07–.22, p = 0.02). • Participants in the treatment group were 1.84 times more likely to be included in a lower pain intensity category compared with participants in the generic information treatment group (cum OR = 1.84, 95%CI 1.04–3.25, *p* = 0.035). • Both physical and mental quality of life improved more in the treatment group than in the generic information treatment group (p’s < 0.05). • NSSD between groups on the recovery index (RI-10).
	Web-based generic information related to the surgery	Low	Low	**✓**	**✕**	**✕**	**✕**	**✕**	**✕**	**✕**	**✕**	**✕**	**✕**	

*Notes*: *CBT* Cognitive Behavioural Therapy, *ACT* Acceptance and Commitment Therapy, *NSSD* non statistically significant difference, *h* hour/s; ? = unclear or cannot determine

#### Intervention intensity

Intensity of RTW programs was determined by the frequency and duration of the intervention (see Table 1 for definitions). Where it was possible to determine the frequency and duration of the interventions, we discuss results below. It was not possible to comment on the effect of intervention intensity in 10 RCTs, as they compared interventions of the same intensity [25, 27, 28, 30, 31, 34, 35, 37, 38, 42].

In two RCTs, a low-to-moderate intensity intervention was compared with a high intensity intervention [26, 38]. In one of these RCTs, when comparing a low intensity health guidance intervention with either (1) a tailored physical activity program plus health guidance or (2) a chronic pain self-management program plus health guidance, there was no statistically significant difference between participants on measures of RTW, work ability, pain, and kinesiophobia (fear of movement) [26]. In the second RCT, there was also no difference in RTW between participants in a high intensity psychological disorder-focused CBT intervention compared with participants in a moderate intensity RTW-focused CBT intervention [38]. While participants in the high intensity psychological disorder-focused CBT intervention experienced superior reduction of psychiatric symptoms from pre- to post-treatment, participants in the RTW-focused CBT intervention experienced greater reduction in psychiatric symptoms from post-treatment to 12 months follow up [38]. One RCT compared a high intensity individual work-focused CBT intervention with a no-treatment comparison group [32]. In this study there was no statistically significant difference between participants on measures of RTW or mental health.

In eight RCTs, participants in an intervention were compared to individuals who were receiving care as usual (CAU) [30, 31, 33, 36, 37, 39–41]. CAU was generally quite broad and varied across studies, including: a disability evaluation [30]; rehabilitation in a standard care facility [31]; ‘usual care’ provided by regular health contacts [33]; functional restoration [36]; standard preoperative surgery-related information and post-operative physical rehabilitation [37]; cardiac rehabilitation and as-needed counselling [39]; contact with a range of health professionals [40, 41], as well as treatment for mental health problems in some cases [40] and regular consultations with occupational physicians [41]. In 3 of these RCTs, there was no statistically significant difference between CAU or intervention participants on primary outcome measures of RTW [37, 39, 40]. Furthermore, in the same studies there was no statistically significant difference between CAU or intervention participants on secondary outcomes measuring catastrophising [37], fear avoidance [37], pain [37], disability scores [37], work ability [39], mental quality of life [39], or depressive symptoms [40]. In another study, participants in the CAU intervention groups experienced similar outcomes overall; however, when stratifying participants by psychological disorder diagnosis, some between-group differences in sickness absence days, work ability, and psychological symptoms were observed [31].

The remaining 4 RCTs showed results in favour of intervention groups over CAU groups. Participants in a rehabilitation-oriented coaching program showed a higher RTW than those who received CAU (disability evaluation with no medical advice) at 1-year follow up [30]. Eight per cent of participants in the CAU group did not return to work, compared to 4% in the coaching program [29]. Similarly, overall RTW was higher in an intervention group receiving motivational interviewing (21.6%) compared to a CAU group receiving functional restoration (9.5%) [36]. In another study, participants in a multi-disciplinary intervention were more likely to self-report increased working hours and work-related engagement compared to participants in a “usual care” group, but participants in an ACT intervention were not [33]. In the last of these RCTs, participants in a CAU group had a slower RTW than participants in a tailored e-Health program (media*n* = 77 versus 50 days, *p* = 0.03), but there was no statistically significant between-group difference in full RTW [41].

#### Intervention characteristics

Specific characteristics of each intervention in the included RCTs are shown in Table 3. An aggregate summary of these characteristics is also displayed in Table 4. A broad analysis of extracted characteristics from the 42 intervention/comparison groups across the 18 RCTs shows that the inclusion of (1) a RTW focus, (2) behavioural activation, and (3) psychoeducation, were most common across the RTW interventions.

**Table 4:** Counts of specific characteristics used in the interventions / comparison groups (*n* = 42) in the included RCTs (*n* = 18)

	Intervention Characteristics									
	Early Timing	Multi-Disciplinary	RTW Focus	Exposure	Cognitive Restructuring	Behavioural Activation	Goal Setting	Values Clarification	Problem Solving	Psychoeducation
**Yes**	14	17	25	15	13	28	10	9	14	24
**No**	24	21	12	21	22	8	25	26	21	11
**Unclear**	4	4	5	6	7	6	7	7	7	7
**Overall (%)**	33	40	**60**	36	31	**67**	24	21	33	**57**

### Reviews (n = 7)

General study information, intervention characteristics, and main findings of the included reviews are shown in Table 5*.* All included reviews were systematic and four included meta-analysis. The reviews generally aimed to broadly evaluate the effectiveness of RTW interventions, except for one study which aimed to investigate the effectiveness of particular intervention characteristics [1]. The total number of studies included in each of the reviews ranged from seven to 42 (153 studies in total), and they were mostly controlled quantitative designs. Four RCTs included in the current systematic review [29, 33, 34, 41] were also represented in two of the included reviews [3, 13], representing an overlap in 3% of studies. Target populations in the included reviews were individuals with mental health issues (1 review), work-related PTSD (1 review), musculoskeletal issues (*n* = 1 review), cancer (2 reviews), and other health problems resulting in sick leave (2 reviews).

**Table 5:** General study information, intervention characteristics, and findings from included reviews

Author (year)	Type of review	Objective	Study Types	Participants	Findings
**de Boer, Taskila** **[**2**]**	Systematic review & meta-analysis	Evaluate the effectiveness of RTW interventions	15 RCTs	Adults with cancer diagnosis and employed (*n* = 1835)	Low quality evidence that psycho-educational interventions are equivalent to CAU. Moderate quality evidence that multi-disciplinary interventions are superior to CAU.
**Fong, Murphy** **[**43**]**	Systematic review & meta-analysis	Evaluate the effectiveness of RTW interventions	8 RCTs, 4 quasi-experimental	Adults with current or past cancer diagnosis (*n* = 2151)	Multi-component interventions, including one or more behavioural, psychological, educational or vocational component appear to improve employment status for cancer patients, but high risk of bias in the literature means results should be interpreted with caution. Methodological limitations make isolating specific components difficult.
**Hoefsmit, Houkes** **[**1**]**	Systematic review	Investigate intervention characteristics that facilitate RTW	18 quantitative, 5 systematic reviews	Adults on sick leave for any reason (*n* unclear)	Generally, early timed (within 6-weeks of initial sick leave) and multi-disciplinary RTW interventions are effective. For musculoskeletal-related sick leave, time-contingent and activating (e.g., gradual RTW) interventions are effective.
**Mikkelsen and Michael** **[**3**]**	Systematic review & meta-analysis	Investigate the effectiveness of RTW interventions	31 RCTs, 8 controlled trials	Adults on sick leave with common psychological, stress, somatoform, or personality disorders (*n* = 38,938)	Timing, duration, gradual RTW, and therapeutic elements had no consistent effect. Interventions with workplace contact and multiple components were effective. Interventions targeting stress disorders were effective. Effect sizes / improvements were small.
**Palmer, Harris** **[**44**]**	Systematic review	Investigate the effectiveness of RTW interventions	34 RCTs, 8 cohort studies	Adults on sick leave with musculoskeletal disorders (*n* unclear)	Early timed and low duration interventions were effective. Interventions that included gradual RTW were effective. Effects were only modest. The other characteristics were ineffective.
**Stergiopoulos, Cimo** **[**45**]**	Systematic review	Investigate the effectiveness of RTW interventions	1 systematic review, 3 RCTs, 3 pre-post	Adults on sick leave with work-related PTSD (*n* = 212, in 6 original studies)	There was good preliminary evidence for the effectiveness of exposure in RTW interventions.
**Vogel, Schandelmaier** **[**13**]**	Systematic review & meta-analysis	Investigate the effectiveness of RTW interventions compared to CAU	14 RCTs	Adults on sick leave or with disability (*n* = 12,568)	Review found that RTW programs had no effects compared to usual practice on RTW outcomes.

*Note*: *CAU* care as usual

^a^Intervention characteristics were only included if they were relevant to the current study (i.e., components of low/medium intensity psychosocial interventions).

^b^Feedback referred to interventions that involved psychological screening followed by individual feedback (e.g., coping strategies) in the case of positive diagnosis.

#### Mental health issues

Two included reviews reported on the effectiveness of RTW program characteristics for individuals with mental health issues. The systematic review and meta-analysis by Mikkelson and Michael [3] included 39 studies (a total of 38,938 participants) and looked at the effectiveness of RTW interventions for adults on sick leave with common psychological, stress, somatoform, or personality disorders. Findings from this review suggested that interventions with workplace contact, multiple components, and targeting stress disorders were effective for RTW; however, effect sizes/improvements were small. The review also reported that timing, duration, gradual RTW, and therapeutic elements of RTW interventions had no consistent effect. The systematic review by Stergiopolous and colleagues [45] was more specific, focussing solely on adults on sick-leave due to work-related PTSD. This review included one systematic review and six original studies, totalling 212 participants. Findings from this review suggested that there is good preliminary evidence for the effectiveness of exposure techniques in RTW interventions for individuals on sick-leave due to work-related PTSD.

#### Musculoskeletal issues

One systematic review, including 42 studies, reported on the effectiveness of RTW interventions for adults on sick leave with musculoskeletal disorders [44]. Findings from this review suggested that interventions which are timed early, low in duration, and include gradual RTW were effective for individuals with musculoskeletal issues; however, effect sizes were only modest.

#### Cancer

Two systematic reviews with meta-analyses reported on the effectiveness of RTW program characteristics for individuals with cancer. The systematic review and meta-analysis by de Boer and colleagues [2] included 15 RCTs (a total of 1835 participants) and evaluated the effectiveness of RTW interventions for employed adults with a cancer diagnosis. Findings from this review suggested that psycho-educational interventions are equivalent to care-as-usual in terms of RTW; however, the quality of evidence drawn upon was quite low, meaning this finding may be unreliable. When drawing upon moderate quality RCTs only, the review found that multi-disciplinary interventions were superior to care-as-usual for adults with a cancer diagnosis. The systematic review and meta-analysis by Fong and colleagues [43] included 12 studies (a total of 2151 participants) and evaluated the effectiveness of RTW interventions for adults with a current or past cancer diagnosis. Similar to findings by de Boer and colleagues [2], this review reported that multi-component interventions, including one or more behavioural, psychological, educational, or vocational component appeared to improve employment status for cancer patients; however, again, high risk of bias in the available literature means results should be interpreted with caution.

#### Other sick leave

Two systematic reviews reported on the effectiveness of RTW interventions for individuals on sick leave for any reason. The systematic review by Hoefsmit and colleagues [1] aimed to investigate specific intervention characteristics that facilitate RTW. This review included 18 quantitative studies and five systematic reviews. Findings from this review suggest that, early timed (within 6-weeks of initial sick leave) and multi-disciplinary RTW interventions were generally effective. More specifically, for musculoskeletal-related sick leave, time-contingent and activating (e.g., gradual RTW) interventions were effective. The systematic review (and meta-analysis) by Vogel and colleagues [13] aimed to investigate the effectiveness of RTW interventions, compared to care-as-usual, for adults on sick leave or with disability. The review included 14 RCTs, with a total of 12,568 participants. The review found that RTW programs had no effects compared to usual practice on RTW outcomes.

### Grey literature (*n* = 5)

Information about the grey literature documents and recommendations are shown in Table 6*.* Target populations included people with psychological problems (*n* = 2), people with any injury or illness (*n* = 2), and people who had experienced a motor vehicle accident (*n* = 1). All injuries and illnesses of the target populations were work-related. The four most recent published documents (Comcare, 2014; CPT Insurance Regulator, 2018; Safe Work Australia, 2018; Worksafe Tasmania, 2018) have based their recommendations at least to some extent on the earliest document by Comcare (2012), which appears to be a central model for RTW processes in Australia.

**Table 6:** Document information and recommendations for RTW from included grey literature

Author	Year	Title	Target Population	Relevant Recommendations	Source
Comcare	2012	Clinical Framework for the Delivery of Health Services	People on sick leave due to any work-related injury or illness	Measure and demonstrate effectiveness of treatment (e.g., track modifiable factors such as depression, use valid and reliable measures); provide education about nature of injury/illness and psychoeducation about cognitive-behavioural models of wellbeing; encourage maintenance of activity in all life domains; address unhelpful beliefs related to fear-avoidance, catastrophising, lack of acceptance, low self-efficacy, blame, and perception of injustice; facilitate self-management through techniques such as collaborative goal setting, pacing, relaxation, exposure); implement SMART (specific, measurable, achievable, relevant, timed) goals focused on optimising function, participation, and RTW; use evidence-based treatments	Comcare (2012)
Comcare	2014	Working for Recovery: Suitable employment for return to work following psychological injury	People on sick leave due to work-related psychological problems	Respond early; perform a detailed assessment; clarify work capacity; identify suitable duties; identify participant strengths; promote activation at home, work, and in community; use problem solving strategies; create a RTW plan including goal setting; organise gradual RTW; address maladaptive beliefs about pain and injury; develop healthy coping strategies; increase perceived control; address relapse prevention	Comcare (2014)
CTP Insurance Regulator	2018	SA CTP Framework for Injury Recovery and Early Intervention	People on sick leave who have experienced motor vehicle trauma	Intervene early; focus on person, not the injury; measure and demonstrate the effectiveness of treatment (e.g., outcome measures about goals or work status); address unhelpful beliefs (e.g., about pain and treatment expectancies); increase engagement in activities at home and work as soon as possible; provide education about the nature of the injury; facilitate a self-management plan; create SMART goals; use evidence-based treatment	CTP Insurance Regulator (2018)
Safe Work Australia	2018	Taking Action: A best practice framework for the management of psychological claims in the Australian workers’ compensation sector	People on sick leave due to work-related psychological problems	Provide early intervention (within 3-months of initial sick leave); focus on worker; use collaborative care; problem solve barriers to RTW; encourage worker to pursue RTW opportunities; engage in follow-up contact with worker (face-to-face or via telephone) to discuss milestones and turning points; use plain English in documentation; assess and align worker expectations; screen for biopsychosocial risk factors (e.g., health conditions, financial stress); establish a review and evaluation process based on agreed goals; use an explicit work-focus; target and improve RTW expectancies of worker (e.g., with motivational interviewing); use complimentary contact modes of telephone and web-based delivery to prevent delays or if worker lives rurally	Safe Work Australia (2018)
Worksafe Tasmania	2018	Managing workplace injuries in Tasmania: A handbook for primary treating medical practitioners	People on sick leave due to any work-related injury or illness	Measure and demonstrate the effectiveness of treatment (e.g., track progress); address psychosocial barriers such as unhelpful beliefs and coping strategies, financial insecurity, low motivation; optimise expectations of worker (e.g., beliefs in recovery); promote benefits of remaining active (e.g., maintaining normal activities); focus on worker strengths; provide education about injury/illness and treatment; use SMART goals focused on function and RTW; promote healthy living habits (e.g., good diet, exercise, sleep, relaxation); promote a pacing approach of graded exposure to activities; use evidence-based treatment	Worksafe Tasmania (2018)

#### RTW guidelines for individuals with work-related psychological problems

Comcare (2014) and Safe Work Australia (2018) provide RTW guidelines for individuals on sick leave due to work-related psychological problems. Both of these guidelines recommend that in assisting RTW for individuals with work-related psychological problems, the response or intervention must be: (1) early; (2) use problem solving strategies; (3) include planning, goal setting, and progress reviews; (4) target and improve work capacities and identify suitable duties; and, (5) encourage the worker to pursue/activate RTW.

#### RTW guidelines for individuals with work-related injury, illness, or motor vehicle trauma

Comcare (2012), CPT Insurance Regulator (2018), and Worksafe Tasmania (2018) provide RTW guidelines for individuals on sick leave due to work-related injury, illness, or motor vehicle trauma. Overall, guidelines from the three sources are very similar and recommend that in assisting RTW for individuals with work-related injury, illness, or motor vehicle trauma, interventions should: (1) use evidence-based treatments; (2) provide education about the injuries/illnesses and psychoeducation about cognitive-behavioural models of wellbeing; (3) encourage maintenance or increase of activities (e.g., at home or work); (4) facilitate self-management using pacing and graded exposure to activities; (5) address unhelpful beliefs (e.g., fear-avoidance, catastrophizing, and low self-efficacy); (6) use SMART goals (particularly to facilitate RTW); and, (7) measure and demonstrate effectiveness of treatments/interventions by tracking progress.

## Discussion

This systematic review sought to determine what constitutes an effective RTW intervention. Eighteen RCTs and seven reviews (all moderate-to-high quality) were included. Most studies came from central or northern Europe, and target populations primarily included individuals with musculoskeletal problems and psychological problems. RTW success was primarily considered in terms of employment status, sickness absence, work-related engagement levels, and disability/insurance claims; however, secondary outcomes including psychological symptoms, pain, and quality of life were also considered, which is a strength of the review. Interventions were typically well-known structured psychosocial interventions, including CBT and ACT. Key findings are discussed below.

The first key finding from the systematic review is that RTW interventions are worthwhile as they appear to help people get back to work, with the limited evidence suggesting that participating in specified RTW programs may be superior for RTW outcomes than receiving care as usual (CAU). Four RCTs comparing RTW interventions with CAU showed results in favour of the intervention groups [30, 33, 36, 41], while another four RCTs found similar RTW outcomes between individuals participating in a RTW program and those receiving CAU [31, 37, 39, 40]. It is important to note that CAU was quite extensive in some cases, which may mean the effects of some RTW interventions were potentially underestimated. For example, in regard to participants who were receiving CAU, studies reported that these individuals actually had relatively high levels of engagement with health care providers such as psychologists or occupational physicians, and were often involved in physical therapies, pain management, psychological therapies, and counselling. In examining the intervention descriptions (Tables 4 & 5), it was not possible to determine whether ‘usual care’ from health practitioners was more intensive than the interventions being assessed. If control participants did engage in higher intensity treatments than intervention participants, the effects of the interventions would be underestimated considerably. Future studies should try to avoid this issue by including detailed reporting of study methodologies [43], explicitly specifying CAU activities, and attempting to avoid contamination.

The reviews included in the current systematic review largely mirror results from the included RCTs, suggesting that RTW interventions are effective for individuals with mental health issues [3, 45], musculoskeletal problems [44], cancer [2], and other health issues [1], but also pointing out potential limitations across the available literature. For example, the Mikkelsen and Michael [3] and Palmer, Harris [44] reviews reported RTW effect sizes to be small-modest, while the review by de Boer, Taskila [2] found that RTW interventions were superior to CAU only when considering moderate quality RCTs (e.g., excluding low quality RCTs). Similarly, the review by Fong, Murphy [43] found that RTW interventions were better than CAU but also highlight the high risk of bias in the literature. Given the overall quality of a study predicts the obtained effect size, there is a need to interpret such literature with caution [9, 46].

The second key finding from the systematic review is that low-to-moderate intensity interventions may potentially be a good place to start, given the costs and resources required are less than that of higher intensity interventions which appear to provide limited additional benefits. Where it was possible to determine the intensity of RTW interventions (based on the frequency and duration of intervention contact), we explored whether low-to-moderate intensity interventions could yield similar results to high intensity RTW programs. In two RCTs a low-to-moderate intensity intervention was compared with a high intensity intervention. In one of these studies, there was no statistically significant difference between participants on measures of RTW, work ability, pain, and kinesiophobia (fear of movement), when comparing a low intensity health guidance intervention with either a tailored physical activity intervention plus health guidance, or with a chronic pain self-management intervention plus health guidance [26]. In the other study, there was also no difference in RTW outcomes between participants in a high intensity psychological disorder-focused CBT intervention compared with participants in a moderate intensity RTW-focused CBT intervention [38]. These studies, along with the other RCTs which found very few statistically significant differences in RTW outcomes for individuals in interventions of varying intensities, provide preliminary support for a stepped-approach to RTW interventions starting with low-to-moderate intensity. However, future research should work towards understanding around whether different sub-groups, or more complex RTW clients, would benefit from varying intervention intensities.

The third key finding from the systematic review is that several common characteristics were consistently seen across effective RTW interventions. Across the studies included in our systematic review, a great breadth and variety of intervention characteristics were observed. A broad analysis of these characteristics showed that the application of a RTW Focus, Behavioural Activation, and Psychoeducation were most common across the RTW interventions. Given the relative success of all interventions presented in our systematic review, these may be potentially important characteristics for ensuring successful RTW program outcomes. However, more rigorous research is needed to confirm the specific role of these characteristics in potentially contributing to an intervention’s success. Common mental disorders and musculoskeletal problems are the leading cause of sickness absence in high-income countries [3, 4, 7, 8]. This was represented in our systematic review, with the majority of participant samples including individuals with either psychological issues [25, 29, 31–33, 35, 38, 40, 41] or musculoskeletal problems [25, 26, 28, 30, 33, 34, 36] which affected their work participation. According to a number of meta-analyses, psychoeducation [47–49] and behavioural activation [50–54] can contribute to improved health outcomes for individuals experiencing musculoskeletal pain and mental health difficulties. As such, the application of these strategies to these populations is considered to be appropriate. Psychoeducation and Behavioural Activation are also relatively inexpensive, easy to implement, and broadly acceptable to people [48], making them good foundational strategies when designing low-to-moderate intensity RTW interventions.

### Practical recommendations

The reciprocal nature of work and psychological distress has been emphasised through disability claims in high income countries [9, 10], as well as the AUD 11.8 billon in productivity losses associated with poor mental health every year in Australia [55]. Given therapeutic elements like problem solving, goal setting, and cognitive restructuring are suggested to be both under-represented in, and important for, RTW interventions in the current review, this may outline an initial step forward in the RTW space. According to Comcare and Safe Work Australia guidelines, it is important that evidence-based RTW interventions include a focus on (1) early intervention, (2) therapeutic CBT-based strategies (e.g., psychoeducation, problem solving, goal setting, behavioural activation, graded exposure, pacing, and cognitive restructuring), and (3) regular measurement of progress. While results of the current review showed that a RTW focus was predominant across interventions (60%), along with behavioural activation (67%) and psychoeducation (57%), a greater focus on other therapeutic strategies may be required to further improve RTW interventions moving forward. Despite being highlighted as important in the grey literature, strategies like problem solving, goal setting, graded exposure, pacing, cognitive restructuring, and values clarification were underrepresented in the reviewed interventions (between 0 and 36%).

Low intensity cognitive behaviour therapy (LICBT) offers an existing evidence-based approach provided by trained behavioural health coaches that can be adapted for a RTW population, containing all the psychological aspects suggested to be important by the peak Australian RTW bodies. Originating in the UK, the efficacy, utility, and flexibility of LICBT programs targeting high prevalence mental health disorders are well documented, with recovery rates reported to between 49 and 62% [56]. Recovery in LICBT programs is defined as the movement of those who were initially in caseness, to below caseness as measured by the psychometric measures used. LICBT programs and workbooks have been developed for varying nationalities around the world, including an Australian population [56], all of which include common elements such as psychoeducation, goal setting, and the regular measurement of psychological distress levels. Moreover, these programs and workbooks have specific focuses on behavioural activation, problem solving, graded exposure, pacing, cognitive restructuring, or worry management. Therefore, an opportunity may exist to ensure effective RTW interventions, by modifying existing LICBT support and resources to meet recommendations outlined by peak Australian RTW organisations, ensuring support is provided by trained behavioural health coaches (absent in 14 of the 18 studies in the current review), and increasing access to support via non-traditional platforms.

### Limitations

Findings from this systematic review should be considered within the constraints of some methodological limitations. A meta-analysis of the included studies could not be performed due to considerable heterogeneity in RTW interventions, samples used, and the assessment of RTW outcomes across studies. These diverse elements impeded our ability to synthesise the evidence and draw reliable conclusions. Methodological weaknesses of some included studies – especially the relatively high amount of service-provision identified amongst CAU groups and limited details provided on specific intervention characteristics in some RCTs – made it difficult to determine the true effectiveness of RTW interventions and to make recommendations for specific characteristics which should be included in RTW interventions. Despite the common use of psychoeducation and behavioural activation strategies across effective RTW interventions, and their known benefits for individuals with psychological or musculoskeletal problems, it is not possible to confirm the specific role these characteristics may have played in contributing to an intervention’s success. Furthermore, RTW may also be affected by other factors, such as labour market characteristics, which were not considered in this review. Most studies included in the systematic review were conducted in central and northern Europe, as such research across a wider range of contexts is needed. However, according to the grey literature included in our systematic review, several of the intervention characteristics used in the included studies are also recommended by peak bodies for RTW interventions in the Australian context, reflecting likely generalisability of results.

## Conclusion

The results of this systematic review highlight the positive role psychosocial RTW interventions can play in helping people with a range of issues, from psychological to musculoskeletal problems, get back to work. The available evidence suggests that a low intensity approach to RTW interventions may be an appropriate first option before investment in more intensive and arguably more expensive approaches, as the latter appear to provide limited additional benefits. Based on the interventions scrutinized in this systematic review, and recommendations by peak Australian bodies, foundational strategies which could be used in such an intervention include those offered by a low intensity cognitive behaviour therapy framework. Despite the utility of information summarized in this systematic review, there is a need for more high-quality, rigorous RCTs to assist in providing reliable evidence to make specific recommendations about creating effective psychosocial RTW programs.

## Supplementary Information

**Additional file 1.** Peer-Reviewed Literature Search Terms.

## Data Availability

Data sharing is not applicable to this article as no datasets were generated or analysed during the current study. All relevant information is included in the manuscript.
