# Supplementary material for: Determining what constitutes an effective psychosocial ‘return to work’ intervention: a systematic review and narrative synthesis
Source: BMC Public Health. 2021 Nov 25;21:2164. doi: 10.1186/s12889-021-11898-z (PMC8620530; doi:10.1186/s12889-021-11898-z)
Supplement: Supplementary file 1 — Additional file 1. Peer-Reviewed Literature Search Terms. [file 12889_2021_11898_MOESM1_ESM.docx]

# Supplementary File 1

## **Peer-Reviewed Literature Search Terms**

Database(s): **Ovid MEDLINE(R) and Epub Ahead of Print, In-Process & Other Non-Indexed Citations, Daily and Versions(R)** 1946 to July 23, 2019

| **#** | **Searches** | **Results** |
| --- | --- | --- |
| 1 | "return to work".ti,ab,kw. | 8649 |
| 2 | Return to Work/ | 2088 |
| 3 | ("work reintegration" or "vocational reintegration" or "return to employment").ti,ab. | 270 |
| 4 | 1 or 2 or 3 | 9523 |
| 5 | (case management or case manager or absence management or assertive community treatment or management program* or therapy or therapeutic or treatment or intervention).ti,ab. | 5712301 |
| 6 | ((project or program* or model or interview? or plan? or measure* or intervention* or evaluation or evaluate or rehabilitation or management or reintegration or "face to face" or "one on one" or in person or personal* or customi?ed or tailored or co ordinat* or coordin* or multi?disciplinary or multi?disciplinary or Multi?modal or integrated or patient centred or patient focused or occupational or vocational or counselling or individual?) and ("on the job" or work focused or work related or workplace or work location or work?site or work place or job site or worksite)).ti,ab. | 41931 |
| 7 | (rehabilitation and (project or program* or plan* or model* or intervention* or interview)).ti,ab. | 62576 |
| 8 | exp Occupational Health Services/ | 10425 |
| 9 | exp Disability Evaluation/ | 50290 |
| 10 | Rehabilitation, Vocational/ | 9316 |
| 11 | Program Evaluation/ | 59960 |
| 12 | 5 or 6 or 7 or 8 or 9 or 10 or 11 | 5857871 |
| 13 | 4 and 12 | 6186 |
| 14 | limit 13 to (english language and yr="2011 -Current") | 2915 |

**CINAHL**

| **#** | **Searches** | **Results** |
| --- | --- | --- |
| 1 | TX "return to work" |  |
| 2 | (MH "Job Re-Entry") |  |
| 3 | TX ("work reintegration" or "vocational reintegration" or "return to employment") |  |
| 4 | S1 OR S2 OR S3 |  |
| 5 | TX (case management or case manager or absence management or assertive community treatment or management program* or therapy or therapeutic or treatment or intervention) |  |
| 6 | TX ((project or program* or model or interview? or plan? or measure* or intervention* or evaluation or evaluate or rehabilitation or management or reintegration or "face to face" or "one on one" or in person or personal* or customi?ed or tailored or co ordinat* or coordin* or multi?disciplinary or multi?disciplinary or Multi?modal or integrated or patient centred or patient focused or occupational or vocational or counselling or individual?) and ("on the job" or work focused or work related or workplace or work location or work?site or work place or job site or worksite)) |  |
| 7 | TX (rehabilitation and (project or program* or plan* or model* or intervention* or interview)) |  |
| 8 | (MH "Occupational Health Services+") |  |
| 9 | (MH "Disability Evaluation+") |  |
| 10 | (MH "Rehabilitation, Vocational") |  |
| 11 | (MH "Program Evaluation") |  |
| 12 | S5 OR S6 OR S7 OR S8 OR S9 OR S10 OR S11 |  |
| 13 | S4 AND S12 |  |
| 14 | ***NOTE: have to manually put in limits of English language and 2011-2019*** | 3262 |

**PsycInfo**

| **#** | **Searches** | **Results** |
| --- | --- | --- |
| 1 | "return to work".ti,ab,id. | 2443 |
| 2 | Reemployment/ | 1377 |
| 3 | ("work reintegration" or "vocational reintegration" or "return to employment").ti,ab. | 117 |
| 4 | 1 or 2 or 3 | 2926 |
| 5 | (case management or case manager or absence management or assertive community treatment or management program* or therapy or therapeutic or treatment or intervention).ti,ab. | 889417 |
| 6 | ((project or program* or model or interview? or plan? or measure* or intervention* or evaluation or evaluate or rehabilitation or management or reintegration or "face to face" or "one on one" or in person or personal* or customi?ed or tailored or co ordinat* or coordin* or multi?disciplinary or multi?disciplinary or Multi?modal or integrated or patient centred or patient focused or occupational or vocational or counselling or individual?) and ("on the job" or work focused or work related or workplace or work location or work?site or work place or job site or worksite)).ti,ab. | 38500 |
| 7 | (rehabilitation and (project or program* or plan* or model* or intervention* or interview)).ti,ab. | 27241 |
| 8 | exp Occupational Health/ | 3644 |
| 9 | exp Disability Evaluation/ | 522 |
| 10 | Vocational Rehabilitation/ | 5889 |
| 11 | Program Evaluation/ | 12255 |
| 12 | 5 or 6 or 7 or 8 or 9 or 10 or 11 | 945949 |
| 13 | 4 and 12 | 1821 |
| 14 | limit 13 to (english language and yr="2011 -Current") | 834 |

**SCOPUS**

| **#** | **Searches** | **Results** |
| --- | --- | --- |
| 1 | TITLE-ABS-KEY("return to work") |  |
| 2 | TITLE-ABS ( "back to work" OR "back-to-work" ) |  |
| 3 | TITLE-ABS ("work reintegration" or "vocational reintegration" or "return to employment") |  |
| 4 | #1 OR #2 OR #3 |  |
| 5 | TITLE-ABS ( "case management" OR "case manager" OR "absence management" OR "assertive community treatment" OR "management program*" OR therapy OR therapeutic OR treatment OR intervention ) |  |
| 6 | TITLE-ABS ((“project or program*” or model or interview? or plan? or measure* or intervention* or evaluation or evaluate or rehabilitation or management or reintegration or "face to face" or "one on one" or “in person” or personal* or customi*ed or tailored or co ordinat* or coordin* or multi*disciplinary or multi*disciplinary or Multi*modal or integrated or “patient centred” or “patient focused” or occupational or vocational or counselling or individual?) and ("on the job" or “work focused” or “work related” or workplace or “work location” or work*site or “work place” or “job site” or worksite)) |  |
| 7 | TITLE-ABS (rehabilitation and (project or program* or plan* or model* or intervention* or interview)) |  |
| 8 | TITLE-ABS ( "Occupational Health Services" ) |  |
| 9 | TITLE-ABS (“Disability Evaluation”) |  |
| 10 | TITLE-ABS (“Vocational Rehabilitation”) |  |
| 11 | TITLE-ABS (“Program Evaluation”) |  |
| 12 | #5 or #6 or #7 or #8 or #9 or #10 or #11 |  |
| 13 | #4 and #12 |  |
| 14 | ( LIMIT-TO ( PUBYEAR , 2019 ) OR LIMIT-TO ( PUBYEAR , 2018 ) OR LIMIT-TO ( PUBYEAR , 2017 ) OR LIMIT-TO ( PUBYEAR , 2016 ) OR LIMIT-TO ( PUBYEAR , 2015 ) OR LIMIT-TO ( PUBYEAR , 2014 ) OR LIMIT-TO ( PUBYEAR , 2013 ) OR LIMIT-TO ( PUBYEAR , 2012 ) OR LIMIT-TO ( PUBYEAR , 2011 ) ) AND ( LIMIT-TO ( LANGUAGE , "English" ) ) | 3471 |

**Scopus search string:**

( ( TITLE-ABS-KEY ( "return to work" ) )  OR  ( TITLE-ABS ( "back to work"  OR  "back-to-work" ) )  OR  ( TITLE-ABS ( "work reintegration"  OR  "vocational reintegration"  OR  "return to employment" ) ) )  AND  ( ( TITLE-ABS ( "case management"  OR  "case manager"  OR  "absence management"  OR  "assertive community treatment"  OR  "management program*"  OR  therapy  OR  therapeutic  OR  treatment  OR  intervention ) )  OR  ( TITLE-ABS ( ( "project or program*"  OR  model  OR  interview?  OR  plan?  OR  measure*  OR  intervention*  OR  evaluation  OR  evaluate  OR  rehabilitation  OR  management  OR  reintegration  OR  "face to face"  OR  "one on one"  OR  "in person"  OR  personal*  OR  customi*ed  OR  tailored  OR  co  AND ordinat*  OR  coordin*  OR  multi*disciplinary  OR  multi*disciplinary  OR  multi*modal  OR  integrated  OR  "patient centred"  OR  "patient focused"  OR  occupational  OR  vocational  OR  counselling  OR  individual? )  AND  ( "on the job"  OR  "work focused"  OR  "work related"  OR  workplace  OR  "work location"  OR  work*site  OR  "work place"  OR  "job site"  OR  worksite ) ) )  OR  ( TITLE-ABS ( rehabilitation  AND  ( project  OR  program*  OR  plan*  OR  model*  OR  intervention*  OR  interview ) ) )  OR  ( TITLE-ABS ( "Occupational Health Services" ) )  OR  ( TITLE-ABS ( "Disability Evaluation" ) )  OR  ( TITLE-ABS ( "Vocational Rehabilitation" ) )  OR  ( TITLE-ABS ( "Program Evaluation" ) ) )  AND  ( LIMIT-TO ( PUBYEAR ,  2019 )  OR  LIMIT-TO ( PUBYEAR ,  2018 )  OR  LIMIT-TO ( PUBYEAR ,  2017 )  OR  LIMIT-TO ( PUBYEAR ,  2016 )  OR  LIMIT-TO ( PUBYEAR ,  2015 )  OR  LIMIT-TO ( PUBYEAR ,  2014 )  OR  LIMIT-TO ( PUBYEAR ,  2013 )  OR  LIMIT-TO ( PUBYEAR ,  2012 )  OR  LIMIT-TO ( PUBYEAR ,  2011 ) )  AND  ( LIMIT-TO ( LANGUAGE ,  "English" ) )

**ProQuest**

| **#** | **Searches** | **Results** |
| --- | --- | --- |
| 1 | ab,ti("return to work") |  |
| 2 | ab,ti( "back to work" OR "back-to-work" ) |  |
| 3 | ab,ti("work reintegration" or "vocational reintegration" or "return to employment") |  |
| 4 | S1 OR S2 OR S3 |  |
| 5 | ab,ti( "case management" OR "case manager" OR "absence management" OR "assertive community treatment" OR "management program*" OR therapy OR therapeutic OR treatment OR intervention ) |  |
| 6 | ab,ti((project or program* or model or interview? or plan? or measure* or intervention* or evaluation or evaluate or rehabilitation or management or reintegration or "face to face" or "one on one" or in person or personal* or customi?ed or tailored or co ordinat* or coordin* or multi?disciplinary or multi?disciplinary or Multi?modal or integrated or patient centred or patient focused or occupational or vocational or counselling or individual?) and ("on the job" or work focused or work related or workplace or work location or work?site or work place or job site or worksite)) |  |
| 7 | ab,ti(rehabilitation and (project or program* or plan* or model* or intervention* or interview)) |  |
| 8 | ab,ti( "Occupational Health Services" ) |  |
| 9 | ab,ti(“Disability Evaluation”) |  |
| 10 | ab,ti(“Vocational Rehabilitation”) |  |
| 11 | ab,ti(“Program Evaluation”) |  |
| 12 | S5 or S6 or S7 or S8 or S9 or S10 or S11 |  |
| 13 | S4 and S12 |  |
| 14 | ***NOTE: have to manually put in limits of English language and 2011-2019*** | 1827 |

**Copy 5: Google Scholar – searched on 9.8.19 (search resulted in identification of two further unique references)**

((“return to work” OR “back to work” OR “job re-entry” OR “return to employment” OR "work reintegration" OR "vocational reintegration") AND ("case management" OR "case manager" OR "absence management" OR "assertive community treatment" OR "management program" OR therapy OR therapeutic OR treatment OR intervention OR project OR program OR model OR interview OR plan OR measure OR intervention OR evaluation OR evaluate OR rehabilitation OR management OR reintegration OR "face to face" OR "one on one" OR "in person" OR personal OR custom OR tailored OR coordinate OR coordinator OR “multi disciplinary” OR “multi modal” OR integrated OR "patient centred" OR "patient focused" OR occupational OR vocational OR counselling OR individual OR "on the job" OR "work focused" OR "work related" OR workplace OR "work location" OR “work site” OR "work place" OR "job site" OR rehabilitation OR "Occupational Health Services" OR "Disability Evaluation” OR "Vocational Rehabilitation" OR "Program Evaluation”)) AND (RCT* OR experiment* OR "control group*" OR random* OR effect* OR comparison* OR efficacy* OR review))

## **Grey Literature Search Terms**

- All these words (one precise search term for each main concept): “return to work” guidelines
- Any of these words (important synonyms for above words): “occupational rehabilitation” OR “return-to-work” OR recommendations OR framework OR “best practices”
- English
- Region: Australia
- File type: PDFs
